# Coinfections with Bacteria, Fungi, and Respiratory Viruses in Patients with SARS-CoV-2: A Systematic Review and Meta-Analysis

**DOI:** 10.3390/pathogens10070809

**Published:** 2021-06-25

**Authors:** Saad Alhumaid, Abbas Al Mutair, Zainab Al Alawi, Abeer M. Alshawi, Salamah A. Alomran, Mohammed S. Almuhanna, Anwar A. Almuslim, Ahmed H. Bu Shafia, Abdullah M. Alotaibi, Gasmelseed Y. Ahmed, Ali A. Rabaan, Jaffar A. Al-Tawfiq, Awad Al-Omari

**Affiliations:** 1 Administration of Pharmaceutical Care, Al-Ahsa Health Cluster, Ministry of Health, Al-Ahsa 31982, Saudi Arabia; 2 Research Center, Almoosa Specialist Hospital, Al-Ahsa 36342, Saudi Arabia; abbas4080@hotmail.com (A.A.M.); g.yousif@almoosahospital.com.sa (G.Y.A.); 3 College of Nursing, Princess Norah Bint Abdul Rahman University, Riyadh 11564, Saudi Arabia; 4 School of Nursing, University of Wollongong, Wollongong, NSW 2522, Australia; 5 Division of Allergy and Immunology, College of Medicine, King Faisal University, Al-Ahsa 31982, Saudi Arabia; zalalwi@kfu.edu.sa; 6 Department of Pharmacy, King Fahad Hofuf Hospital, Al-Ahsa 36441, Saudi Arabia; aalshuui@moh.gov.sa (A.M.A.); saaalomran@moh.gov.sa (S.A.A.); 7 Department of Pharmacy, Maternity and Children Hospital, Al-Ahsa 36422, Saudi Arabia; msalmuhanna@moh.gov.sa (M.S.A.); aaalmuslim@moh.gov.sa (A.A.A.); 8 Department of Pharmacy, Alomran Hospital, Al-Ahsa 36355, Saudi Arabia; abushafia@moh.gov.sa; 9 Department of Pharmacy, Prince Sultan Cardiac Center, Al-Ahsa 36441, Saudi Arabia; aalotaibi274@moh.gov.sa; 10 Molecular Diagnostic Laboratory, Johns Hopkins Aramco Healthcare, Dhahran 31311, Saudi Arabia; arabaan@gmail.com; 11 Infectious Disease Unit, Specialty Internal Medicine, Johns Hopkins Aramco Healthcare, Dhahran 31311, Saudi Arabia; jaffar.tawfiq@jhah.com; 12 Infectious Disease Division, Department of Medicine, Indiana University School of Medicine, Indianapolis, IN 46202, USA; 13 Infectious Disease Division, Department of Medicine, Johns Hopkins University School of Medicine, Baltimore, MD 21287, USA; 14 College of Medicine, Alfaisal University, Riyadh 11533, Saudi Arabia; awad.omari@drsulaimanalhabib.com; 15 Research Center, Dr. Sulaiman Al Habib Medical Group, Riyadh 11372, Saudi Arabia

**Keywords:** SARS-Cov-2, co-infection, coinfection, COVID-19, concurrent, bacterial, fungal, viral, meta-analysis

## Abstract

Background: Coinfection with bacteria, fungi, and respiratory viruses in SARS-CoV-2 is of particular importance due to the possibility of increased morbidity and mortality. In this meta-analysis, we calculated the prevalence of such coinfections. Methods: Electronic databases were searched from 1 December 2019 to 31 March 2021. Effect sizes of prevalence were pooled with 95% confidence intervals (CIs). To minimize heterogeneity, we performed sub-group analyses. Results: Of the 6189 papers that were identified, 72 articles were included in the systematic review (40 case series and 32 cohort studies) and 68 articles (38 case series and 30 cohort studies) were included in the meta-analysis. Of the 31,953 SARS-CoV-2 patients included in the meta-analysis, the overall pooled proportion who had a laboratory-confirmed bacterial infection was 15.9% (95% CI 13.6–18.2, n = 1940, 49 studies, *I^2^* = 99%, *p <* 0.00001), while 3.7% (95% CI 2.6–4.8, n = 177, 16 studies, *I^2^* = 93%, *p <* 0.00001) had fungal infections and 6.6% (95% CI 5.5–7.6, n = 737, 44 studies, *I^2^* = 96%, *p <* 0.00001) had other respiratory viruses. SARS-CoV-2 patients in the ICU had higher co-infections compared to ICU and non-ICU patients as follows: bacterial (22.2%, 95% CI 16.1–28.4, *I^2^* = 88% versus 14.8%, 95% CI 12.4–17.3, *I^2^* = 99%), and fungal (9.6%, 95% CI 6.8–12.4, *I^2^* = 74% versus 2.7%, 95% CI 0.0–3.8, *I^2^* = 95%); however, there was an identical other respiratory viral co-infection proportion between all SARS-CoV-2 patients [(ICU and non-ICU) and the ICU only] (6.6%, 95% CI 0.0–11.3, *I^2^* = 58% versus 6.6%, 95% CI 5.5–7.7, *I^2^* = 96%). Funnel plots for possible publication bias for the pooled effect sizes of the prevalence of coinfections was asymmetrical on visual inspection, and Egger’s tests confirmed asymmetry (*p* values < 0.05). Conclusion: Bacterial co-infection is relatively high in hospitalized patients with SARS-CoV-2, with little evidence of *S. aureus* playing a major role. Knowledge of the prevalence and type of co-infections in SARS-CoV-2 patients may have diagnostic and management implications.

## 1. Introduction

Coronavirus disease 2019 (COVID-19) is caused by the severe acute respiratory syndrome coronavirus 2 (SARS-CoV-2) and was first described in Wuhan, China in 2019. Globally, as of 15 April 2021, there have been 137,866,311 confirmed cases of COVID-19, including 2,965,707 deaths, as reported by the World Health Organization [1]. Coinfection with SARS-CoV-2 and other bacterial, fungal, and respiratory viral pathogens [2,3,4], Gram-positive and Gram-negative bacteria [5,6,7], Middle East respiratory syndrome coronavirus (MERS-CoV) [8], and influenza [9,10,11,12,13] has been described. However, the reported frequency is variable. Such coinfections in patients with SARS-CoV-2 may be a cause of increased morbidity and mortality [2,6,7,14,15,16,17,18,19,20,21,22]. Thus, timely diagnosis is important to initiate appropriate therapy and limit the overuse of antimicrobial agents. Previous studies, including case series [2,5,8,11,14,15,16,19,20,23,24,25,26,27,28,29,30,31,32,33,34,35,36,37,38,39,40,41,42,43,44,45,46,47,48,49,50], cohort studies [3,4,6,7,9,10,12,13,17,18,21,22,51,52,53,54,55,56,57,58,59,60,61,62,63,64,65,66,67,68,69,70], and meta-analyses [71,72,73], have shown variable results. In light of recent studies evaluating coinfections in SARS-CoV-2 patients, we aimed to re-evaluate the prevalence of bacterial, fungal, and respiratory viral coinfections in a comprehensive meta-analysis. Moreover, we aimed to identify the risk-factors, characteristics, and consequences associated with SARS-CoV-2 coinfection.

## 2. Methods

### 2.1. Design

This is a meta-analysis and was conducted per the Preferred Reporting Items for Systematic Reviews and Meta-Analyses [PRISMA] guidelines [74]. We searched PROQUEST, MEDLINE, EMBASE, PUBMED, CINAHL, WILEY ONLINE LIBRARY, and NATURE for full texts. Search keywords included Coronavirus infection OR SARS coronavirus OR severe acute respiratory syndrome OR COVID OR SARS AND mixed infection OR bacterial pneumonia OR bacteremia OR bacterial infection OR fungal infection OR viral infection OR respiratory infection OR mycosis OR coinfect OR co-infect OR concomitant infect OR concurrent infection OR mixed infect OR coinfection OR co-infection. The search included English language studies from 1 December 2019 to 31 March 2021. Then, articles were kept if the title and abstract contained discussion about bacterial, fungal, and/or respiratory viral co-infection in SARS-CoV-2 patients. In addition, we used manual backward snowballing of the bibliographies of retrieved articles to include additional relevant articles.

### 2.2. Inclusion and Exclusion Criteria

The included articles were pertinent if these articles included patients with a positive SARS-CoV-2 reverse-transcription polymerase chain reaction (RT-PCR) test of any age and a described co-infection on presentation or developed during the course of the disease or during hospital stay. These cases were retained if bacteria, fungi, and/or viruses were detected in the respiratory tract or blood culture samples and were excluded if they were identified from other samples. We aimed to include randomized controlled trials, cohort studies, and case series, and excluded other types of studies.

### 2.3. Data Extraction

Three authors (S.A., A.A., and J.A.) reviewed the retrieved studies and chose relevant articles. Data were extracted using key headings as indicated in Table 1. The study designs were classified as well. The extracted information included: authors; study location; study design and setting; publication year; number of SARS-CoV-2 patients tested for co-pathogens; number of coinfected patients; age; proportion of male patients; percentage of patients requiring intensive care unit (ICU) and mechanical ventilation; mortality rates; proportion of patients with bacterial, fungal, and/or respiratory viral coinfections; total organisms identified; antimicrobials prescribed; laboratory techniques for co-pathogen detection; assessment of study risk of bias; and remarks on notable findings.

### 2.4. Quality Assessment

The Newcastle–Ottawa Scale [NOS] was the primary tool for examining the quality of included studies, as described previously [75]. The tool provides maximum scores of 4 for selection, 2 for comparability, and 3 for exposure/outcome. High-quality studies have a score of >7, and moderate-quality studies have a score of 5–7. Quality assessment was performed independently by four authors (A.M.A., S.A.A., G.Y.A., and A.R.) and a consensus was used to resolve any disagreement.

### 2.5. Data Analysis

We examined primarily the proportion of confirmed acute bacterial, fungal and/or respiratory viral infections in patients with SARS-CoV-2. This proportion was further classified based on initial presentation or during the course of the illness. Taking a conservative approach, a random effects with the DerSimoniane–Laird model was used [76], which produces wider confidence intervals [CIs] than a fixed effect model. Results were illustrated using forest plots. The Cochran’s chi-square (*χ^2^*) and the *I^2^* statistic provided the tools of examining statistical heterogeneity [77]. An *I^2^* value of >50% suggested significant heterogeneity [78]. Examining the source of heterogeneity, a subgroup analysis was conducted based on ICU and non-ICU admission or only ICU admission. Funnel plots and Egger’s correlation test estimate publication bias and *p* value < 0.05 indicates statistical significance [79]. R version 4.1.0 with the packages metafor and meta was used for all statistical analyses.

## 3. Results

### 3.1. Characteristics and Quality of Included Studies

**Figure 1:**
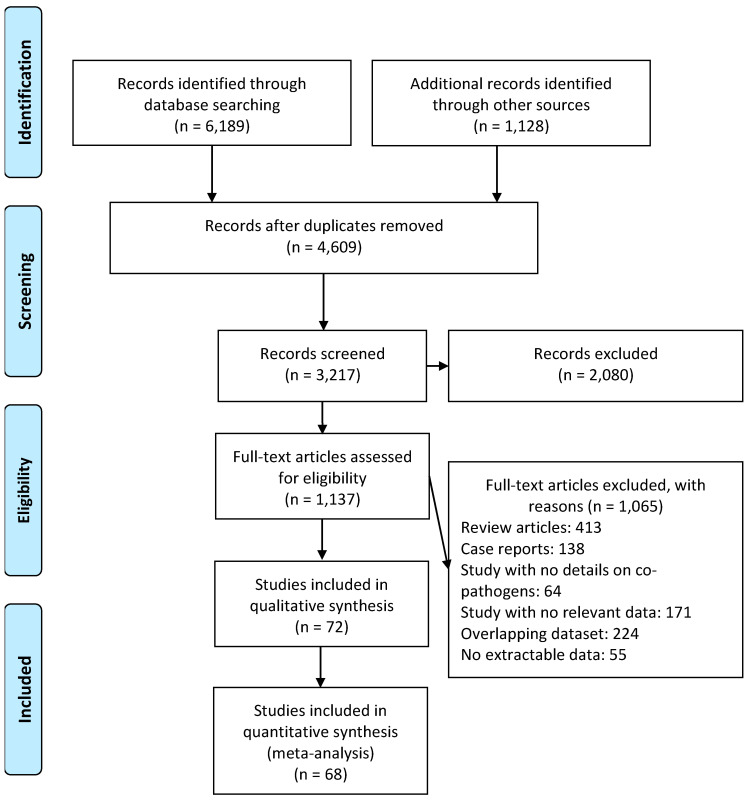
Flow diagram of literature search and data extraction from studies included in the systematic review and meta-analysis.

Of the initial 7317 retrieved publications, there were 4609 duplicate articles, and 2080 articles were found to be irrelevant based on their titles and abstracts and were excluded. An additional 1065 articles were excluded after review, meaning that we included 72 articles in the systematic review [2,3,4,5,6,7,8,9,10,11,12,13,14,15,16,17,18,19,20,21,22,23,24,25,26,27,28,29,30,31,32,33,34,35,36,37,38,39,40,41,42,43,44,45,46,47,48,49,50,51,52,53,54,55,56,57,58,59,60,61,62,63,64,65,66,67,68,69,70,80,81,82], while 68 articles were included in the meta-analysis [2,3,4,5,6,7,8,9,10,11,13,14,15,16,17,18,19,20,21,22,23,24,25,26,27,28,29,30,31,32,33,34,35,36,37,39,40,41,43,44,45,46,47,48,49,50,51,52,53,54,55,56,57,59,60,61,62,63,64,65,66,67,68,69,70,80,81,82] (Figure 1).

The included studies had a total of 31,953 SARS-CoV-2 infected patients as detailed in Table 1. Of those patients, 25,302 (79.2%) were from 32 cohort studies and 20.8% were from 40 case series. The geographical distribution of these studies was as follows: Asia (n = 36), Europe (n = 22), and North America (n = 14). The majority of the studies were single center and only 24 studies were multi-center. Laboratory techniques for co-pathogen detection within studies included 19 that used respiratory samples and RT-PCR tests [4,5,8,11,12,13,29,33,37,38,53,55,58,59,62,63,66,70,80], 17 that used serologic tests (antibodies) [6,10,14,19,24,31,32,35,36,43,44,45,50,52,60,64,68], 15 that used RT-PCR tests with respiratory and/or blood cultures [7,9,17,18,23,26,28,34,39,42,51,56,57,65,67], 12 that did not specify their testing methods [3,15,16,22,25,30,40,41,46,47,49,81], five that only used respiratory and/or blood cultures [2,21,48,54,61], and three that tested both serology and RT-PCR [27,69,82] (Table 1). Seven studies examined patients for *influenza A* and *B* only [10,11,19,41,60,68,70]; while five studies evaluated patients for the presence of *Chlamydia* or *Mycoplasma* [6,24,35,52,82]; and four studies only evaluated for the presence of fungi [17,23,39,42]. The proportion of patients receiving antibiotic agents was reported in 34 studies [2,6,7,14,16,17,18,19,20,21,23,24,31,34,35,36,37,39,40,42,43,45,46,48,49,51,52,56,57,60,64,70,80,82]. The most commonly used antimicrobials were macrolides (n = 355), 2nd/3rd/5th generation cephalosporins (n = 157), fluoroquinolones, (n = 150), antifungals (n = 62), beta-lactams/beta-lactam inhibitors (n = 26), beta-lactams (n = 21), tetracyclines (n = 17), linezolid (n = 13), carbapenems (n = 4), and glycopeptides (n = 2). The median NOS score was 6 with a range from 5 to 8. The NOS quality was moderate for 66 studies, and high quality for 6 studies. The majority (60/72, 83.3%) of the studies included only adult patients. The proportion of male patients had a median of 55.9% [interquartile range (IQR) 48.9–71.9%]. The majority (n = 58) of the studies included any hospitalized patient, and 14 studies included only critically ill. Sixteen, thirteen, and four studies exclusively reported on respiratory viral, bacterial, and fungal co-infections, respectively; and the remaining 39 studies reported on bacterial, fungal, and respiratory viral co-infections; Table 1.

### 3.2. Meta-Analysis of Bacterial, Fungal, and Respiratory Viral Co-Infections in Patients with SARS-CoV-2

**Figure 2:**
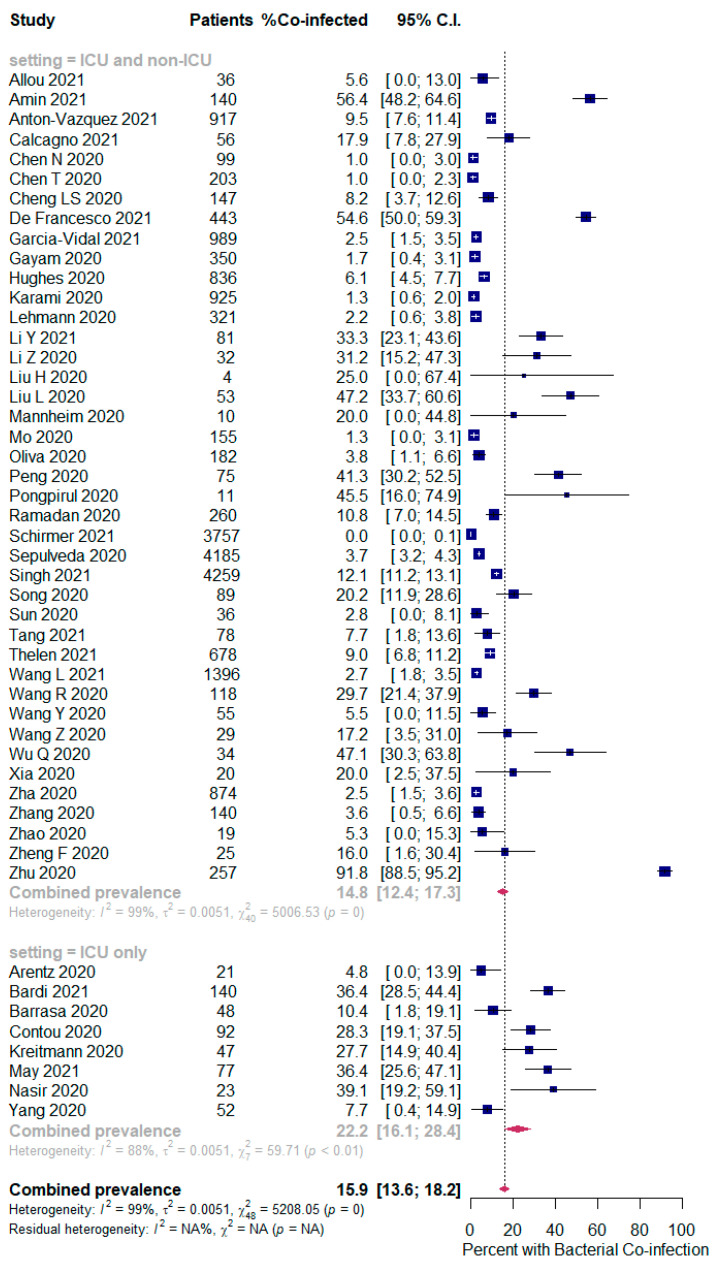
Forest plot of proportion of SARS-CoV-2 patients with bacterial co-infections (all patients in the upper panel and only ICU patients in the lower panel).

**Figure 3:**
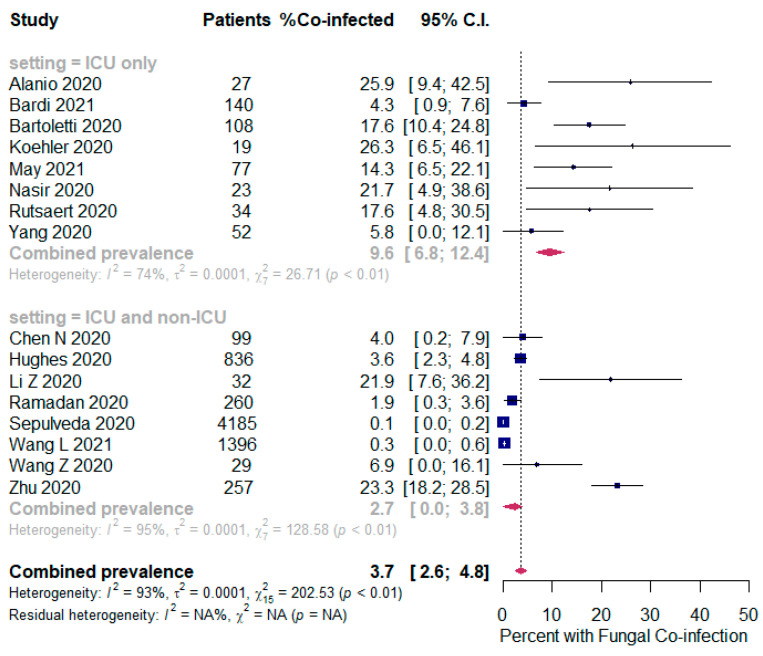
Forest plot of proportion of SARS-CoV-2 patients with fungal co-infections (all patients in the upper panel and only ICU patients in the lower panel).

**Figure 4:**
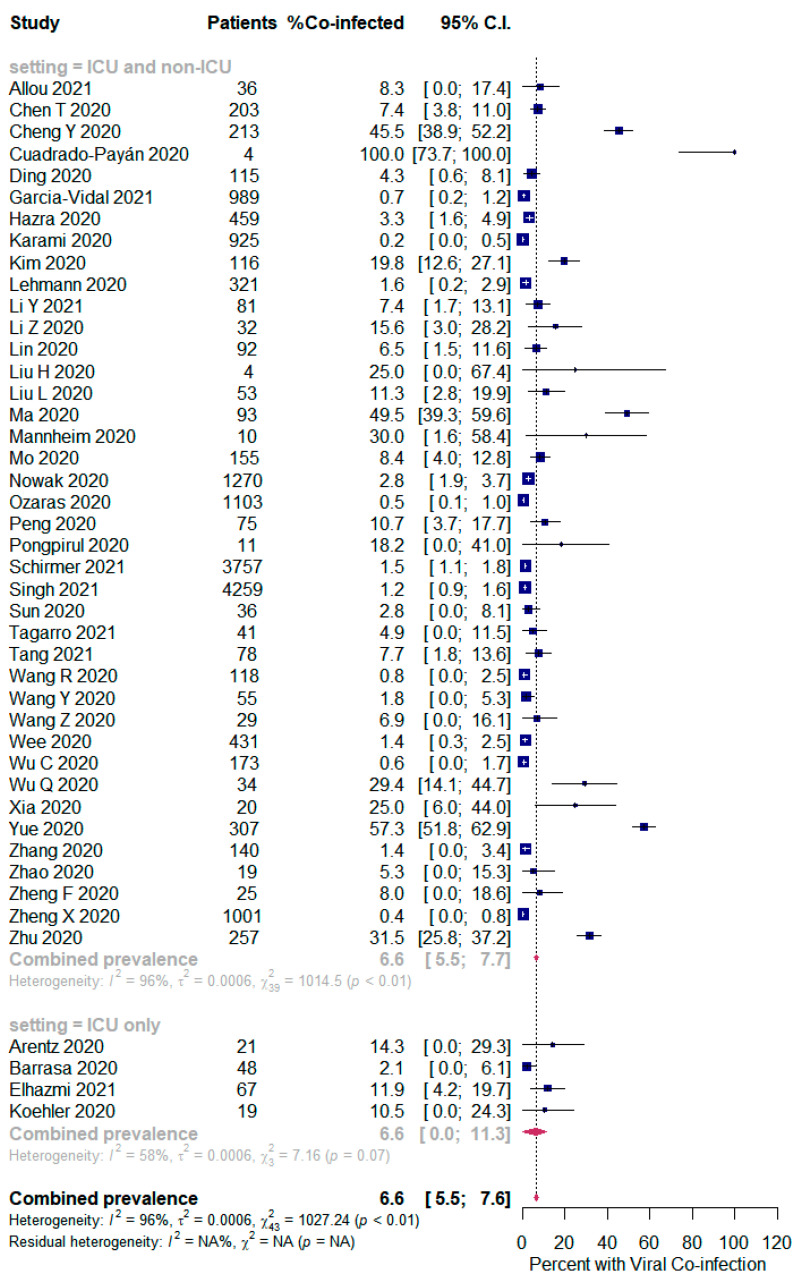
Forest plot of proportion of SARS-CoV-2 patients with respiratory viral co-infections (all patients in the upper panel and only ICU patients in the lower panel).

The overall pooled proportions of SARS-CoV-2 patients who had laboratory-confirmed bacterial, fungal, and respiratory viral coinfections were 15.9% (95% CI 13.6 to 18.2, n = 1940, 49 studies, *I^2^* 99%, *p <* 0.00001), 3.7% (95% CI 2.6 to 4.8, n = 177, 16 studies, *I^2^* 93%, *p <* 0.00001), and 6.6% (95% CI 5.5 to 7.6, n = 737, 44 studies, *I^2^* 96%, *p <* 0.00001), respectively; (Figure 2,Figure 3,Figure 4).

In bacterial coinfected SARS-CoV-2 patients, subgroup analysis showed some difference in the rates between all patients (ICU and non-ICU group); and the ICU only group (14.8% (95% CI 12.4 to 17.3, n = 1802, 41 studies, *I^2^* = 99%); and 22.2% (95% CI 16.1 to 28.4, n = 137, 8 studies, *I^2^* = 88%), respectively); Figure 2. In the fungal co-infected SARS-CoV-2 patients, subgroup analysis showed a significant difference in the rates between all patients (ICU and non-ICU); and ICU only patients [2.7% (95% CI 0.0 to 3.8, n = 155, 8 studies, *I^2^* = 95%); and 9.6% (95% CI 6.8 to 12.4, n = 62, 8 studies, *I^2^* = 74%), respectively]; Figure 3.

However, in the respiratory viral co-infected SARS-CoV-2 patients, subgroup analysis showed an identical proportion between all patients (ICU and non-ICU) and the ICU only patients [6.6% (95% CI 5.5 to 7.7, n = 723, 40 studies, *I^2^* = 96%); and 6.6% (95% CI 0.0 to 11.3, n = 14, 4 studies, *I^2^* = 58%), respectively]; Figure 4.

**Figure 5:**
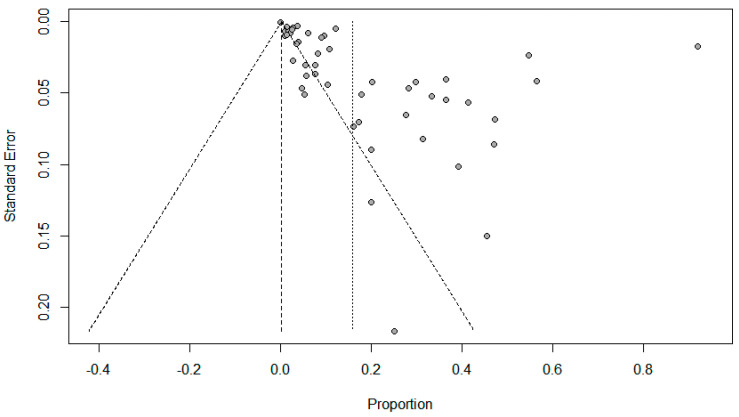
Funnel plots evaluating publication bias for the pooled effect size determining the prevalence of bacterial co-infections in SARS-Cov-2 patients.

**Figure 6:**
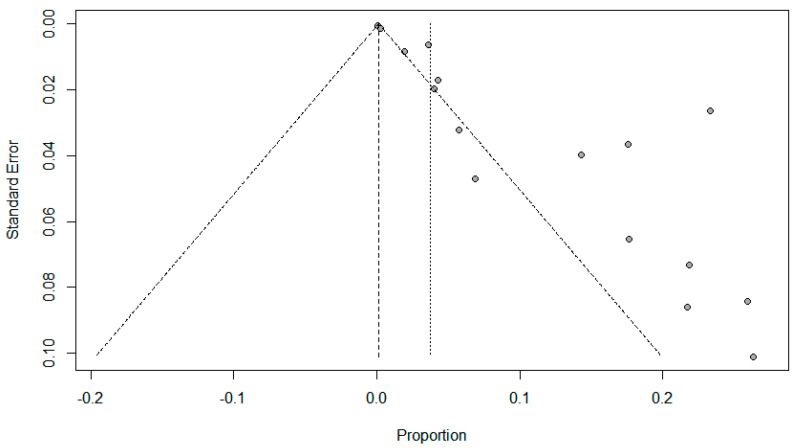
Funnel plots evaluating publication bias for the pooled effect size to determine the prevalence of fungal co-infections in SARS-Cov-2 patients.

**Figure 7:**
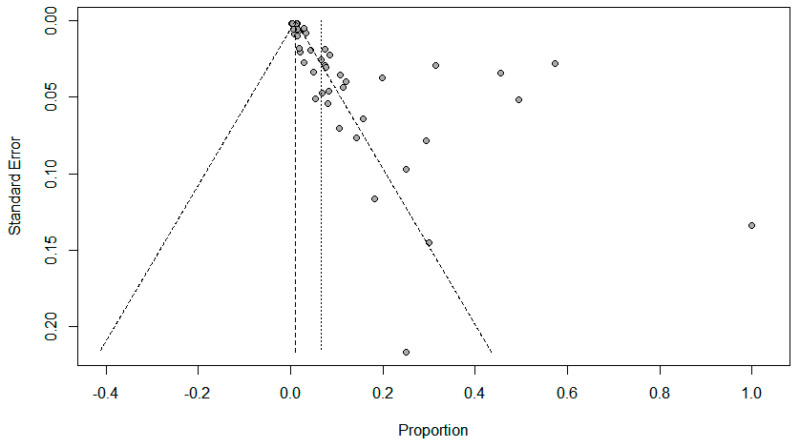
Funnel plots to evaluate publication bias for the pooled effect size to determine the prevalence of other respiratory viral co-infections in SARS-Cov-2 patients.

Funnel plots for possible publication bias for the pooled effect size to determine the prevalence of coinfections in SARS-Cov-2 patients appeared asymmetrical on visual inspection, and Egger’s tests confirmed asymmetry with *p* values < 0.05; Figure 5, Figure 6 and Figure 7.

### 3.3. Bacterial, Fungal and Respiratory Viral Co-Pathogens

Specific bacterial co-pathogens were reported in 49/72 (68%) studies, which is about 57.3% of the reported co-infections. The most common bacteria were *S. aureus* (n = 1095), *M. catarrhalis* (n = 352), *M. pneumoniae* (n = 338), *S. pneumoniae* (n = 316), *C. pneumoniae* (n = 261), *K. pneumoniae* (n = 259), and *H. influenzae* (n = 197) (Table 2).

Fungal co-pathogens were reported in 16/72 (22.2%) studies, which is equal to only 3.2% of the reported co-infections. The most common fungal organisms were *Aspergillus* spp. (n = 68), *Aspergillus fumigatus* (n = 43), Other *Candida* spp. (n = 29), *Candida albicans* (n = 25) and *Aspergillus flavus* (n = 10) (Table 3).

Respiratory viral co-pathogens were reported in 44/72 (61.1%) studies, representing about 39.5% of the reported co-infections. The most common respiratory viruses were EBV (n = 644), HHV6 (n = 574), *Influenza A* virus (n = 355), HMPV (n = 328), and *Adenovirus* (n = 144) (Table 4).

## 4. Discussion

In this large systematic review and meta-analysis, we included 31,953 patients with laboratory-confirmed SARS-CoV-2 from 72 observational studies in order to estimate the prevalence of coinfections with bacterial, fungal, and respiratory viral pathogens. This study showed the following microbial coinfection prevalences: bacterial (15.9%, 95% CI 13.6–18.2); fungal (3.7%, 95% CI 2.6–4.8); and respiratory viral (6.6%, 95% CI 5.5–7.6) coinfections. Bacterial and fungal coinfections were more common in ICU patients ((22.2%%, 95% CI 16.1–28.4) and (9.6%, 95% CI 6.8–12.4), respectively) than mixed ICU and non-ICU patients, as expected. However, respiratory viral co-infection rate in SARS-CoV-2 patients was identical in both groups (6.6%, 95% CI 0.0–11.3). Nevertheless, the included studies in this meta-analysis are case series and cohort studies and we did not identify any randomized controlled trials addressing this issue. In addition, the included studies comprised only admitted patients, which may skew the findings and should not be generalized to all SARS-COV-2 patients. Non-admitted COVID-19 patients were not represented in these studies and thus the exact prevalence of coinfections could not be calculated for all SARS-CoV-2 infected patients [83,84,85]. The findings in this meta-analysis showed different results from previous systematic meta-analyses that evaluated coinfections among COVID-19 patients [71,72,73]. We reported a higher prevalence of coinfections in hospitalized SARS-CoV-2 patients. The current meta-analysis is more comprehensive and included a total of 71 studies [2,4,5,6,7,8,9,10,11,12,13,14,15,16,17,18,19,20,21,22,23,24,25,26,27,28,29,30,31,32,33,34,35,36,37,38,39,40,41,42,43,44,45,46,47,48,49,50,51,52,53,54,55,56,57,58,59,60,61,62,63,64,65,66,67,68,69,70,80] and one abstract [3], including a total of 31,953 patients. The inclusion of 18 recently published studies [2,3,5,6,7,8,9,10,12,13,14,22,24,27,41,62,64,65] contributed to the refinement of the estimate of the pooled prevalence of pathogens contributing to coinfections in SARS-CoV-2 patients.

In this meta-analysis, bacterial coinfection was more prevalent than fungal and other respiratory viruses. This finding may reflect high rates of antimicrobial use for admitted patients with SARS-CoV-2 infection to treat documented or presumed bacterial co-infections. Thus, it is important to study the occurrence, type, and intended antimicrobial agent use in SARS-COV-2 patients in order to develop additional strategies for the optimal use of antimicrobial agents in this population. As expected, bacterial, fungal, and other respiratory viral co-infections in SARS-CoV-2 patients were more frequent in ICUs compared with non-ICU locations [2,20,28,57], a finding which has previously been described in systematic reviews [71,72] and may reflect the epicenter role of ICUs in both infections and antimicrobial resistance. One of the reasons for the increase in infection rate in ICUs could be due to the simultaneous infection of the virus and bacterium. Viruses can facilitate the attachment and colonization of the bacteria in the respiratory tract, which is certainly no exception for SARS-CoV-2 [86]. Nevertheless, other factors such as ICU type, used equipment rate, admission or discharge criteria, high workload or nurse ratio, etc. can also affect the quality of care and the rate of ICU-acquired, healthcare-associated infections [87,88]. With observed strains currently being placed on healthcare systems during the upstroke of the SARS-CoV-2 pandemic, guidelines must focus on the maintenance of good knowledge and compliance of infection prevention and control [89], antimicrobial stewardship [90], and robust surveillance for healthcare-associated infections and antimicrobial resistance [91,92].

The most common method used to detect co-infections in the studies included in this review was RT-PCR tests for respiratory samples. The choice of diagnostic test for pathogens depends in part upon test availability and how soon the results are needed. If available, molecular assays (RT-PCR or, alternatively, a rapid molecular assay) are preferred over antigen detection tests (e.g., direct and indirect immunofluorescence assays) because molecular tests are the most sensitive [93]. Nevertheless, positive RT-PCR tests might indicate recently resolved infection or colonization [94,95]. In addition, many studies evaluated serological (antibodies) tests with this method detecting co-infections in SARS-CoV-2 patients. Application of serologic laboratory technique for co-pathogens detection across all studies was likely to reveal an even higher overall co-infection proportion than found in our study. Consecutively, it is possible that positive serology indicated recent and not acute infection in included patients [96]. Serologic testing is useful primarily for research purposes and antibody-based tests might produce false negative results during the window period. It is worthwhile to mention that administration of broad-spectrum antimicrobials to a large percentage of the patients included in this review might relatively have lowered the sensitivity of microbial culture methods, which could have resulted in underestimation of the true numbers of co-infections.

Specific co-infecting pathogens in SARS-CoV-2 patients were identified in this study from the 72 included studies. In line with the previous systematic reviews and meta-analyses [71,72], *M. pneumoniae*, *K. pneumoniae*, and *H. influenzae* were among the predominant co-pathogens. However, in this meta-analysis, *S. aureus* was the most common bacterial pathogens co-infecting SARS-CoV-2 patients. However, this finding needs to be carefully interpreted, as 85.6% of all *S. aureus* co-pathogens in our review were reported by one study [58]. *S. aureus* infections are a known complication of other viral pandemics, such as the Spanish flu and the H_1_N_1_ influenza pandemic [97,98]. *S. aureus* is known to act synergistically in SARS-CoV-2 patients, increasing mortality and severity of disease [38,99]. The proposed mechanisms of viral-induced *S. aureus* co-infections include viral modification of airway structures and increased adherence of the organism to respiratory mucosa, as well as initiation of immune-suppressive responses [22,100,101]. Further investigations are necessary to confirm an association between SARS-CoV-2 infection and susceptibility to *S. aureus* coinfections.

It was noted that male patients with SARS-CoV-2 were more likely to have coinfections than female [13]. However, patients with pneumococcal pneumoniae and SARS-CoV-2 were mostly females [24]. Older age appears to be the major risk factor associated with coinfections with bacteria and respiratory viruses [12,38,43,58,62] and fungi [39]. This might be attributed mainly to the differences in the inclusion criteria and the population age groups included in the studies, or it could be explained by the gender-based biological differences in the host immune response to COVID-19 infection [102]. The age-dependent defects in T-cell and B-cell function and the excess production of type 2 cytokines could lead to a deficiency in control of viral replication and more prolonged proinflammatory responses, potentially leading to poorer outcomes [103]. Yet, SARS-CoV-2 patients of any age may develop such coinfections and experience severe disease, especially in those with comorbidities, even in young people [4,53], children [27,49], and infants [40].

A few underlying comorbidities were associated with increased risk of coinfections, and these included obesity [8,12,38], cancer, hepatitis, and kidney disease [12,43]. Laboratory abnormalities that have been described in SARS-CoV-2 patients with bacterial and respiratory viral coinfections were high procalcitonin [47,50,64,80], d-dimer [9], and monocytes [31]; and low neutrophils [31]. Some conclusions could be drawn from available data as to whether patients who have a concurrent bacterial, fungal, and/or respiratory viral infection have a worse prognosis than those in whom SARS-CoV-2 is the only detected pathogen. Mortality in SARS-CoV-2 patients was increased due to bacterial [2,6,14,21], fungal [2,17,20,21], or respiratory viral [20] co-infections compared to SARS-CoV-2 patients with no co-infections. Few studies observed no increase in mortality in COVID-19 patients compared to those who did not have bacterial [3,22,24,35,66], fungal [3,22], or other respiratory viral [66] coinfections. Clinical presentation, laboratory results, radiological findings, and outcome are likely to differ between SARS-CoV-2 positive patients with and without co-infections. Bacterial coinfection increased SARS-CoV-2 patients’ hospital length of stay [18,50], need for ventilatory support [6,28], ARDS [28], shock [28], multi-organ injury [23,32], and caused more severe COVID-19 disease [2,21,28,33,34,53,68]. Two studies reported conflicting results on the role of bacterial [24,36] or respiratory viral [36] coinfection in relation to increasing length of hospital stay or ICU admission [22,24,35]. It was shown that the patterns of SARS-CoV-2 symptoms and clinical outcomes were not different in the bacterial [27] and respiratory viral [10,11,27,66,70] co-infected patients. The severity and time of SARS-CoV-2 disease clearance were not different in patients with respiratory viral co-infections [19,36].

The data on the timing of the occurrence of co-infection was variable. The occurrence of co-infections has a median time of 4–11.5 days (IQR 2–42) of ICU admission [2,17,42]. Bacterial co-infection was infrequent within 2–4 days of hospital admission [22,26]. Nonetheless, considering the high number and severity of bacterial co-infections previously reported in patients with SARS-CoV-2, initiation of antibiotic therapy for all hospitalized patients with COVID-19 is recommended [7]. The approach of administering empiric antibiotic therapy solely to patients who were admitted for SARS-CoV-2 and who presented with a chest X-ray suggestive of bacterial infection, have a need for direct ICU admission, or are severely immunocompromised should be reconsidered. When bacterial co-infection in SARS-CoV-2 patients is suspected, an antibiotic approach with optimal *S. aureus* coverage, such as ceftaroline, ceftriaxone, or cefazolin plus levofloxacin, is recommended in areas with methicillin-sensitive *S. aureus* prevalence [104].

### Limitations

The main limitation of this meta-analysis is that included studies were observational with no randomized controlled trials; and there was no standardized microbiologic testing at specified intervals. In interpreting funnel plots, the different possible reasons for funnel plot asymmetry should be distinguished. Possible sources of asymmetry in funnel plots might be the wide differences between the included populations in the different studies, publication bias and selective outcome and/or analysis reporting, poor methodological design and inadequate analysis, or asymmetry might have occurred by chance. Furthermore, the analysis was limited to the English literature and thus may miss other studies published in other languages.

## 5. Conclusions

Bacterial co-infection is relatively high in hospitalized patients with SARS-CoV-2, with little evidence of *S. aureus* having a major role. Empiric antibiotic therapy should be considered in SARS-CoV-2 patients who present with a chest X-ray suggestive of bacterial infection, the need for direct ICU admission, or a severely immunocompromised condition. Knowledge of the prevalence and type of co-infections in SARS-CoV-2 patients may have diagnostic and management implications.

## Figures and Tables

**Table 1:** Summary of the characteristics of the included studies with evidence on SARS-CoV-2 and bacterial, fungal, and/or respiratory viral co-infections (n = 72), 2020–2021.

Author, Year, Study Location	Study Design, Setting	Number of SARS-CoV-2 Patients Tested for Co-Pathogens, n	Co-Infected Patients, n (%)	Age (Years)	Male, n (%)	Admitted to ICU, n (%)	Mechanical Ventilation, n (%)	Deaths, n (%)	Bacterial Co-Infection, n (%)	Fungal Co-Infection, n (%)	Respiratory Viral Co-Infection, n (%)	Total Organisms, n	Antimicrobials Use, n	Laboratory Techniques for Co-Pathogen Detection	NOS Score	Key Findings
Alanio et al., 2020 [23], France	Prospective case series, single center	27	7 (25.9)	Median (IQR), 63 (43–79)	5 (71.4)	7 (100)	7 (100)	4 (75.1)	-	7 (25.9)	-	7 *Aspergillus fumigatus*	3 Macrolides 2 Antifungals	Culture from respiratory specimens and GM detection in the BAL and serum	7	Death was not related to pulmonary aspergillosis but to bacterial septic shock and organ failure.
Allou et al., 2021 [9], France	Prospective cohort, single center	36	5 (13.9)	Median (IQR), 68 (57–82)	4 (80)	10 (27.8)	2 (5.5)	0	2 (5.5)	-	3 (8.3)	1 *Influenza A* virus 1 *Branhamella catarrhalis* 1 *S. pneumoniae* 1 *H. influenzae* 1 Human Coronavirus 229E 1 *Rhinovirus* 1 MSSA	Not reported	RT-PCR for naopharyngeal specimens [viruses] AND sputum culture [bacteria and fungi]	7	Level of D-dimer was significantly higher in patients with co-infection compared to patients without co-infection (1.36 mg/mL vs. 0.63 mg/mL, *p* = 0.05).
Amin et al., 2021 [14], United States	Retrospective case series, single center	140	79 (56.4)	Mean (SD), 62.3 (16.3)	55 (69.6)	29 (36.7)	26 (32.9)	38 (48.1)	79 (56.4)	-	-	79 *M. pneumoniae*	All patients received antibiotics coverage against *M. pneumoniae*, however, agents were not reported	Serum antibody test (IgM)	6	Death was significantly higher in patients with *M. pneumoniae* co-infection compared to patients without *M. pneumoniae* co-infection (AOR: 2.28, 95% CI: 1.03–5.03).
Anton-Vazquez et al., 2021 [24], Spain	Retrospective case series, single center	917	87 (9.5)	Median (IQR), 68 (27–92)	37 (42.5)	8 (9.2)	Not reported	15 (17.2)	87 (9.5)	-	-	87 *S. pneumoniae*	Third Generation Cephalosporins were prescribed in the great majority of cases	Serum antibody test (IgM, IgG)	6	Co-infected pneumococcal pneumonia patients compared with COVID-19 patients without pneumococcal testing were mostly female (57% vs. 34%, *p* < 0.001). No differences in age, length of stay, admission to ICU, or mortality were found between groups.
Arentz et al., 2020 [15], United States	Retrospective case series, single center	21	4 (19)	Mean (range), 70 (43–92)	11 (52)	21 (100)	15 (71)	11 (52.4)	1 (4.8)	-	3 (14.3)	1 *Pseudomonas* 2 *Influenza A* virus 1 *Parainfluenza* 3 virus	Not reported	Unspecified	8	Study included 21 ICU patients who had a high rate of ARDS and a high risk of death.
Bardi et al., 2021 [2], United States	Retrospective case series, single center	140	57 (40.7)	Median (IQR), 63 (60–68)	47 (82)	57 (100)	56 (98)	31 (54)	51 (36.4)	6 (4.3)	-	18 *Enterococcus faecium* 11 *Enterococcus faecalis* 16 CoNS 14 *P. aeruginosa* 9 MRSA1 *Klebsiella oxytoca* 1 *Serratia marcescens* 1 *Bacteroides* spp. 1 *Candida glabrata* 4 *Candida albicans* 3 *Aspergillus fumigatus* 3 *Stenotrophomonas maltophilia* 2 *A. baumannii* 2 *Enterobacter cloacae* 1 *Aspergillus terreus* 1 *Hafnia alvei* 1 *H. influenzae* 1 MSSA 1 *K. pneumoniae*	53 Third Generation Cephalosporins 53 Macrolides 47 Other antibiotics	Respiratory tracheal aspirate and blood cultures	6	Co-infection occurred a median of 9 days (IQR 5–11) after admission and was significantly associated with the APACHE II score (*p* = 0.02). Co-infection was significantly associated with death (OR 2.7, 95% CI 1.2–5.9, *p* = 0.015) and longer ICU stay (*p* < 0.001).
Barrasa et al., 2020 [16], Spain	Retrospective case series, multi-center	48	6 (12.5)	Median (IQR), 63 (51–75)	27 (56.2)	48 (100)	45 (93.7)	12 (25)	5 (10.4)	-	1 (2.1)	3 *P. aeruginosa* 1 *Enterococcus faecium* 1 *H. influenzae* 1 MRSA	17 Fluoroquinolones 22 Third Generation Cephalosporins 10 Macrolides 9 Linezolid 15 Beta-Lactams	Unspecified	7	Procalcitonin plasma above 0.5 mg/L was associated with 16% vs. 19% (*p* = 0.78) risk of death after 7 days.
Bartoletti et al., 2020 [17], Italy	Prospective cohort, multi-center	108	30 (27.7)	Median (IQR), 63 (57–70)	24 (80)	108 (100)	108 (100)	44 (40.7)	-	19 (17.6)	-	15 *Aspergillus fumigatus* 3 *Aspergillus niger* 1 *Aspergillus flavus*	9 Macrolides 16 Antifungals	Culture from respiratory specimens and GM detection in the BAL and serum	7	Co-infection of aspergillosis occurred after a median of 4 (2–8) days from ICU admission and a median of 14 (11–22) days from SARS-CoV-2 symptom onset. Mortality was higher in ICU patients co-infected with aspergillosis compared to SARS-CoV-2 patients without the fungal co-infection (44% vs. 19%, *p* = 0.002).
Calcagno et al., 2021 [5], Italy	Retrospective case series, single center	56	10 (17.8)	Mean (SD), 63.3 (18)	6 (60)	Not reported	Not reported	Not reported	10 (17.8)	-	-	7 *S. aureus* 2 *H. influenzae* 1 *E. coli* 1 *M. catarrhalis* 1 *Streptococci agalactiae* 1 *K. pneumoniae* 1 *Enterobacter cloacae*	Not reported	RT-PCR of respiratory tract specimens (nasopharyngeal, BAL, BA, and sputum)	7	Phenomena like viral interference, common receptor usage, different inoculum size, or simply resource competition might explain why dual or multiple concurrent viral respiratory infections are rare.
Chen N et al., 2020 [80], China	Retrospective case series, single center	99	5 (5)	Mean (SD), 55.5 (13.1)	67 (67.7)	23 (23)	17 (17)	11 (11)	1 (1)	4 (4)	-	1 *A. baumannii* 1 *K. pneumoniae* 1 *Aspergillus flavus* 1 *Candida glabrata* 3 *Candida albicans*	70 [cephalosporins, quinolones, carbapenems, tigecycline, and linezolid] 15 Antifungals	RT-PCR via throat swab	7	Six (6%) of patients had high procalcitonin levels.
Chen T et al., 2020 [25], China	Retrospective case series, single center	203	17 (8.4)	Median (IQR), 54 (20–91)	108 (53.2)	34 (16.7)	39 (19.2)	26 (12.8)	2 (0.9)	-	15 (7.4)	4 *Parainfluenza* virus 3 RSV 3 *Adenovirus* 2 *Mycoplasma* 2 *Influenza A* virus 3 *Influenza B* virus	Not reported	Unspecified	7	Two mortality cases were reported in co-infected patients.
Cheng L et al., 2020 [18], Hong Kong	Prospective cohort, single center	147	12 (8.2)	Median (IQR), 49 (30–61)	9 (75)	1 (8.3)	Not reported	0	12 (8.2)	-	-	3 *H. influenzae* 8 MSSA 1 *P. aeruginosa* 1 *S. pneumoniae*	46 Penicillins & cephalosporins 14 Tetracyclines 3 Fluoroquinolones 3 Macrolides	RT-PCR of respiratory tract specimens AND sputum and blood cultures	6	Co-infected SARS-CoV-2 patients had longer length of hospitalization (median: 20 days vs. 27 days, *p* = 0.016).
Cheng Y et al., 2021 [10], China	Prospective cohort, single center	213	97 (45.5)	Median (IQR), 61 (50–68)	47 (48.5)	Not reported	2 (2.1)	3 (3.1)	-	-	97 (45.5)	97 *Influenza A* virus	Not reported	Serum antibody test (IgM)	6	Similar symptoms and clinical outcomes were seen in the SARS-CoV-2 co-infected group compared to the SARS-CoV-2 group without co-infection. Co-infection with *Influenza A* virus had no effect on disease outcome.
Contou et al., 2020 [51], France	Prospective cohort, single center	92	26 (28)	Median (IQR), 61 (55–70)	73 (79)	92 (100)	83 (90)	45 (49)	26 (28)	-	-	10 MSSA 7 *H. influenzae* 6 *S. pneumoniae* 5 *Enterobacteriaceae* 2 *P. aeruginosa* 1 *M. catarrhalis* 1 *A. baumannii*	14 Third Generation Cephalosporins 14 Beta-Lactam/Beta-Lactamase Inhibitors 6 Beta-Lactams 5 Others antibiotics	RT-PCR for respiratory specimens [viruses] AND respiratory and blood cultures [bacteria and fungi]	7	Resistance by co-pathogens to 3^rd^ generation cephalosporin and to amoxicillin–clavulanate combination was observed in 8% and 21%, respectively.
Cuadrado-Payán et al. [11]. 2020, Spain	Retrospective case series, single center	4	4 (100)	Mean (SD), 67 (14.5)	3 (75)	3 (75)	3 (75)	0	-	-	4 (100)	3 *Influenza A* virus 2 *Influenza B* virus	None	RT-PCR for respiratory specimens	7	Clinical courses in co-infected SARS-CoV-2 patients did not differ from those previously reported.
De Francesco et al., 2021 [6], Italy	Retrospective cohort, multi-center	443	242 (54.6)	Mean (SD), 71 (19)	173 (71.4)	Not reported	16 (6.8)	Not reported	242 (54.6)	-	-	242 *C. pneumoniae* 63 *M. pneumoniae*	138 Macrolides	Serum antibody test (IgM, IgG)	6	SARS-CoV-2 co-infected patients were more critical than SARS-CoV-2 patients without co-infection (13.2% vs. 5.9%, *p* = 0.01). Need for ventilatory support was significantly higher in co-infected patients than in only SARS-CoV-2 positive patients (nasal canula: 18.1% vs. 3.6%, *p* < 0.0001; high flow oxygen support: 45% vs. 23.3%, *p* < 0.0001; and non-invasive ventilation: 14.7% vs. 4.6%, *p* = 0.001, respectively). Higher mortality was observed in SARS-CoV-2 patients with *M.* and/or *C. pneumoniae* (24.2% vs. 21.8%, *p* = 0.63).
Ding et al., 2020 [19], China	Retrospective case series, single center	115	5 (4.3)	Mean (SD), 50.20 (9.83)	2 (40)	0	0	0	-	-	5 (4.3)	3 *Influenza A* virus 2 *Influenza B* virus	Five patients received antibiotics; however, agents were not reported.	Influenza serology	7	SARS-CoV-2 co-infected patients did not show severe disease compared to SARS-CoV-2 without *influenza* co-infection (similar laboratory results, imaging, and prognosis). Nasal blockade and pharyngeal pain were more in the SARS-CoV-2 con-infected group.
Elhazmi et al., 2021 [8], Saudi Arabia	Retrospective case series, multi-center	67	8 (11.9)	Mean (SD), 44.4 (11.8)	6 (75)	67 (100)	7 (87.5)	3 (37.5)	-	-	8 (11.9)	8 MERS-CoV	None	RT-PCR for respiratory specimens	7	Seven (87.5%) patients were obese.
Garcia-Vidal et al., 2021 [7], Spain	Retrospective cohort, single center	989	31 (3.1)	Median (IQR), 63 (54.5–74)	18 (58.1)	8 (25.8)	Not reported	5 (16.1)	25 (2.5)	-	7 (0.7)	12 *S. pneumoniae* 7 *S. aureus* 2 *H. influenzae* 1 *M. catarrhalis* 2 *P. aeruginosa* 1 *E. coli* 1 *K. pneumoniae* 1 *Enterococcus faecium* 1 *Proteus mirabilis* 1 *Citrobacter koseri* 6 *Influenza A* virus 3 *Influenza B* virus 1 RSV 1 HSV	26 Macrolides 24 Third Generation Cephalosporins 2 Fifth Generation Cephalosporins	RT-PCR for respiratory specimens [viruses] AND blood, pleural fluids, sputum cultures [bacteria and fungi]	7	Co-infection at COVID-19 diagnosis is uncommon. Worse clinical outcomes were seen in SARS-CoV-2 co-infected patients.
Gayam et al., 2020 [52], United States	Retrospective cohort, single center	350	6 (1.7)	Mean (SD), 57 (10.6)	2 (33.3)	1 (16.7)	1 (16.7)	1 (16.7)	6 (1.7)	-	-	6 *M. pneumoniae*	6 Third Generation Cephalosporins 3 Macrolides 3 Tetracyclines	Serum antibody test (IgM, IgG)	6	Only one patient (16.7%) required ICU admission and experienced organ failure and death.
Hashemi et al., 2021 [12], Iran	Retrospective cohort, multi-center	105 dead patients	Not reported	Range (0 to >60)	Males were > females	Not reported	Not reported	105 (100)	-	-	Not reported	18 *Influenza* virus (H_1_N_1_) 9 *Bocavirus* 8 RSV 5 *Influenza* virus (non-H_1_N_1_) 4 *Parainfluenza* virus 3 HMPV 2 *Adenovirus*	Not reported	RT-PCR for respiratory specimens	5	Most of the co-infected cases were men aged >60 years; and had history of obesity, cancer, hepatitis, and kidney diseases. Prevalence of SARS-CoV-2 and *influenza A* virus co-infection in dead patients was high.
Hazra et al., 2020 [53], United States	Retrospective cohort, single center	459	15 (3.3)	Median, 39	Not reported	Not reported	Not reported	Not reported	-	-	15 (3.3)	2 *Adenovirus* 1 Coronavirus NL63 2 HMPV 3 *Influenza A* virus 1 *Parainfluenza* 2 virus 8 *Rhinovirus*/*Enterovirus*	Not reported	RT-PCR for respiratory specimens	5	Co-infected patients were younger than those only infected with SARS-CoV-2 (age: 39 vs. 58 years, *p* = 0.02).
Hughes et al., 2020 [26], United Kingdom	Retrospective case series, multi-center	836	51 (6.1)	Median (IQR), 69 (55–81)	519 (62)	3 (5.9)	Not reported	Not reported	51 (6.1)	30 (3.6)	-	8 *Enterobacterales* 36 CoNS 4 *Streptococcus* spp. 7 *S. aureus* 4 *Enterococcus* spp. 3 *Candida albicans* 1 *P. aeruginosa* 12 *Pseudomonas* spp. 5 *Enterobacter* spp. 6 *Klebsiella* spp. 2 *Serratia* spp. 24 *Candida* spp. 3 *Aspergillus* spp. 1 *H. influenzae* 1 *Hafnia* spp. 1 *Morganella morganii* 1 *Providencia* spp. 2 *S. maltophilia*	Not reported	RT-PCR for respiratory specimens [viruses] AND blood, sputum, and BAL cultures [bacteria and fungi]	6	Rate of bacterial co-infection in SARS-CoV-2 patients in the early phase of hospital admission was low.
Karami et al., 2020 [54], The Netherlands	Retrospective cohort, multi-center	925	12 (1.2)	Median (IQR), 70 (59–77)	591 (64)	166 (21.9)	Not reported	214 (23.3)	12 (1.2)	-	2 (0.2)	7 *S. aureus* 1 *K. oxytoca* 1 *S. maltophilia* 1 *Parainfluenzae* virus 1 *H. influenzae* 1 *Influenza A* virus 1 *S. pneumoniae* 2 *E. coli*	No extractable data	Blood and sputum cultures [bacteria and viruses]	6	On presentation to the hospital, bacterial co-infections are rare.
Kim et al., 2020 [55], United States	Retrospective cohort, single center	116	23 (19.8)	Median (IQR), 46.9 (14–74)	12 (52.2)	0	0	0	-	-	23 (19.8)	8 *Rhinovirus*/*Enterovirus* 6 RSV 5 Coronavirus (non-SARS, non-MERS) 2 HMPV 1 *Parainfluenza* 1 1 *Parainfluenza* 3 1 *Parainfluenza* 4 1 *Influenza A* virus	Not reported	RT-PCR via nasopharyngeal swab	8	Patients with co-infections did not differ significantly in age (mean, 46.9 years) from those infected with SARS-CoV-2 only (mean, 51.1 years).
Koehler et al., 2020 [20], Germany	Retrospective case series, single center	19	5 (26.3)	Mean (SD), 62.6 (8.8)	3 (60)	5 (100)	Not reported	3 (60)	-	5 (26.3)	2 (10.5)	2 HMPV 5 *Aspergillus fumigatus*	5 Antifungals	RT-PCR for respiratory specimens [viruses] AND GM detection in the BAL and tracheal aspirates	6	Critical cases of SARS-CoV-2 patients were at risk of developing aspergillosis co-infection and had higher mortality.
Kreitmann et al., 2020 [56], France	Prospectivecohort, single center	47	13 (27.6)	Median (IQR), 61 (56–74)	25 (73.5)	47 (100)	Not reported	5 (35.8)	13 (27.6)	-	-	9 *S. aureus* 5 *H. influenzae* 3 *S. pneumoniae* 1 *M. catarrhalis* 1 *Streptococcus agalactiae*	4 Third Generation Cephalosporins 2 Macrolides 3 Other antibiotics	RT-PCR for respiratory specimens and/or cultures	6	Authors argue for initial empirical antibiotic coverage in SARS-CoV-2 patients.
Lehmann et al., 2020 [57], United States	Retrospective cohort, single center	321	12 (3.7)	Mean (SD), 60 (17)	155 (48)	17 (5)	Not reported	22 (7)	7 (2.2)	-	5 (1.5)	2 *S. aureusa* 1 *Proteus mirabilisa* 3 *Influenza A* virus 2 *Rhinovirus*/*Enterovirus* 1 *Bordetella parapertussis* 4 *S. pneumoniae*	Antibiotic use was high (222 [69%]); however, agents were not reported.	RT-PCR for respiratory specimens and/or cultures	7	Community-acquired co-infection in COVID-19 is infrequent and often viral. Co-infection was more common among ICU patients.
Li Y et al., 2021 [27], China	Retrospective case series, single center	81	27 (33.3)	Mean (SD), 76.55 (9.64)	15 (55.6)	1 (3.7)	1 (3.7)	0	27 (33.3)	-	6 (7.4)	23 *M. Pneumoniae* 1 *Influenza A* virus 2 *Influenza B* virus 1 RSV 1 *Adenovirus* 1 *Parainfluenza* virus 3 *M. catarrhalis* 1 *S. pneumoniae*	No extractable data	Direct immunofluorescence test AND serum antibody test (IgM)	7	Almost 1/3 (33.3%) had co-infection. Coinfection did not cause a significant exacerbation in clinical symptoms.
Li Z et al., 2020 [28], China	Retrospective case series, multi- center	32	14 (43.7)	Median (IQR), 57 (47–69)	11 (78.6)	11 (78.6)	4 (28.6)	Not reported	10 (31.2)	7 (21.9)	5 (15.6)	3 *Stephanoascus ciferrii* 4 *Candida albicans* 2 *Staphylococcus epidermidis* 1 *Ralstonia mannitolilytica* 3 *Stenotrophomonas maltophilia* 1 *Bacteroides fragilis* 3 *Burkholderia estoste* 2 *Enterococcus Faecium* 1 *E. coli* 2 *Elizabethkingia meningosepticum* 1 *A. baumannii* 1 RSV 1 HMPV 2 HcoV-HKU1 1 *Rhinovirus* 1 *Parainfluenza* virus 1 *Enterovirus*	Not reported	RT-PCR AND cultres	6	SARS-CoV-2 patients with co-infections were admitted more often to ICU (*p* < 0.05), showed more severe difficulty in breathing (*p* < 0.05), and experienced more complications such as ARDS and shock (*p* < 0.05).
Lin et al., 2020 [29], China	Retrospective case series, single center	92	6 (6.5)	Majority (≈78%) were in the range (18–65)	1:1 ratio	Not reported	Not reported	Not reported	-	-	6 (6.5)	3 RSV 2 *Rhinovirus* 2 HMPV 1 *Parainfluenza* 2 virus 2 HcoV-HKU1	Not reported	RT-PCR of respiratory tract specimens (naso- vs. oropharyngeal source not specified)	7	Limitation of the sensitivity of method for the different respiratory viruses and low load of virus in specimens might have contributed to negative results.
Liu H et al., 2020 [30], China	Retrospective case series, multi-center	4	2 (50)	Range (2 months to 9 years)	1:1 ratio	0	0	0	1 (25)	-	1 (25)	1 *M. pneumoniae* 1 RSV	Not reported	Unspecified	6	Pulmonary involvement was more severe, as simultaneous infection of RSV and SARS-CoV-2 in one child was detected.
Liu L et al., 2020 [31], China	Retrospective case series, single center	53	31 (58.5)	Median (IQR), 38 (28–47)	26 (49)	1 (1.9)	1 (1.9)	0	25 (47.2)	-	6 (11.3)	25 *M. pneumoniae* 2 *Influenza A* virus 2 *Influenza B* virus 2 RSV	25 Fluoroquinolones	Serum antibody test (IgM, IgG)	6	COVID-19 patients co-infected with *M. pneumoniae* had a higher percentage of monocytes (*p* < 0.0044) and a lower neutrophils percentage (*p* < 0.0264).
Ma et al., 2020 [32], China	Retrospective case series, single center	93	46 (49.5)	Median (IQR), 67 (54–72)	51 (54.8)	Not reported	Not reported	44 (47.3)	-	-	46 (49.5)	44 *Influenza A* virus 2 *Influenza B* virus 1 *Adenovirus* 1 *Parainfluenza* virus	Not reported	Serum antibody test (IgM)	6	Critically ill COVID-19 patients with *influenza* were more prone to cardiac injury than those without *influenza*.Critically ill COVID-19 patients with *influenza* exhibited more severe inflammation and organ injury.
Mannheim et al., 2020 [33], United States	Retrospective case series, multi-center	10	4 (40)	Median (IQR), 11 (7–16)	Males were > females	7 (70)	Not reported	0	2 (20)	-	3 (30)	1 *M. pneumoniae* 1 *Adenovirus* 1 *Rhinovirus*/*Enterovirus* 1 *E. coli* 1 *Rotavirus*	Not reported	RT-PCR for respiratory specimens	6	Underlying co-infection might have contributed to severe disease.
Massey et al., 2020 [58], United States	Retrospective cohort, multi-center	1456	Not reported	Mean (SD), 72.4 (20.9)	Not reported	Not reported	Not reported	Not reported	Not reported	-	Not reported	937 *S. aureus* 576 EBV 574 HHV6 328 *M. catarrhalis* 64 *K. pneumoniae* 305 HMPV109 *Adenovirus*	Not reported	RT-PCR for respiratory specimens	6	Advanced age and nursing home status were associated with higher co-infection rates in SARS-CoV-2 patients. In SARS-CoV-2 patients, 86.3% had at least one co-infection compared to 75.7% in the negative SARS-CoV-2 group (*p* < 0.0001).
May et al., 2021 [3], United Kingdom	Retrospective cohort, single center	77	39 (50.6)	Not reported	Not reported	39 (100)	Not reported	Not reported	28 (36.4)	11 (14.3)	-	12 *S. aureus* 1 *Staphylococcus lugdunensis* 7 *H. influenza* 2 *S. pneumoniae* 10 *Klebsiella* spp. 3 *Serratia marcescens* 3 *Citrobacter* spp. 3 *Enterobacter cloacae* 3 *Proteus mirabilis* 2 *E. coli* 2 *P. aeruginosa* 1 *Hafnia alvei* 4 *Enterococcus* spp. 5 *Aspergillus*	Not reported	Unspecified	5	There was no significant correlation between hospital mortality and isolation of a pathogen in early or any respiratory sample (*p* = 0.512 and *p* = 1.0, respectively).
Mo et al., 2020 [81], China	Retrospective cohort, single center	155	12 (7.7)	Median (IQR), 54 (42–66)	86 (55.5)	37 (23.9)	36 (23.2)	22 (14.2)	2 (1.3)	-	13 (8.4)	3 *Parainfluenza* virus 3 RSV 3 *Adenovirus* 2 *Mycoplasma* 2 *Influenza A* virus 2 *Influenza B* virus	Not reported	Unspecified	5	COVID-19 patients were divided into general and refractory groups.
Nasir et al., 2020 [34], Pakitstan	Retrospective case series, single center	23	9 (39.1)	Median (IQR), 71 (51–85)	7 (77.8)	23 (100)	2 (22.2)	4 (17.4)	9 (39.1)	5 (21.7)	-	2 *Aspergillus fumigatus* 1 *Aspergillus niger* 6 *Aspergillus flavus* 2 *P. aeruginosa* 1 *K. pneumoniae* 1 MRSA 2 *Acinetobacter* spp. 1 *Clostridium perfringens* 2 *Stenotrophomonas maltophilia*	7 Macrolides5 Antifungals	Culture from respiratory specimens and GM detection in the BAL, tracheal aspirates and serum	6	Invasive aspergillosis is a complication in moderate to severe COVID-19 patients.
Nowak et al., 2020 [59], United States	Retrospective cohort, multi-center	1204 1270 1103 1103 1103 1103	1 (0.1) 4 (0.3) 17 (1.5) 8 (0.7) 4 (0.4) 2 (0.2)	Mean, 60.1	16 (44)	Not reported	Not reported	Not reported	-	-	36 (2.8)	1 *Influenza A* virus 4 RSV 17 Other Coronaviridae [7 NL63, 5 HKU1, 4 229E, 1 OC43] 8 *Rhinovirus*/*Enterovirus* 4 HMPV 2 *Adenovirus*	Not reported	RT-PCR for respiratory specimens	6	Study hypothesized that competitive advantage may play a role in the SARS-CoV-2 interaction with other respiratory viruses during co-infection.
Oliva et al., 2020 [35], Italy	Retrospective case series, single center	182	7 (3.8)	Median (IQR), 73 (45–79)	4 (57.1)	1 (14.3)	Not reported	0	7 (3.8)	-	-	5 *C. pneumoniae* 2 *M. pneumoniae*	7 Macrolides 1 Teicoplanin 1 Beta-Lactam/Beta-Lactamase Inhibitors 1 Third Generation Cephalosporins	Serum antibody test (IgM)	6	ICU admission and mortality were similar in the SARS-CoV-2 patients co-infected with *M. pneumoniae* or *C. pneumoniae* compared to SARS-CoV-2 group without the co-infection (14.2% vs. 13.7% and 0% vs. 14.2%, respectively).
Ozaras et al., 2020 [60], Turkey	Retrospective cohort, multi-center	1103	6 (0.54)	Mean (SD), 40.5 (14)	3 (50)	0	0	0	-	-	6 (0.5)	2 *Influenza A* virus 4 *Influenza B* virus	6 Macrolides	Direct immunofluorescence test	6	Cases reported in this study were mild to moderate in severity.
Peng et al., 2020 [36], China	Retrospective case series, single center	75	42 (56)	Mean (range), 6.06 years (1 month–15 years)	44 (58.67)	Not reported	Not reported	0	31 (41.3)	-	8 (10.7)	28 *M. pneumonia* 1 *M. catarrhalis* 1 *S. aureus* 1 *S. pneumoniae* 3 *Influenza B* virus 1 *Influenza A* virus 2 *Adenoviridae* 1 CMV 1 RSV	30 Macrolides Thirty-seven patients received antibiotics; however, agents were not reported.	Serum antibody test (IgM)	6	Co-infection never increased patients’ length of stay or decreased time of SARS-CoV-2 virological clearance.
Pongpirul et al., 2020 [37], Thailand	Retrospective case series, multi-center	11	11 (100)	Median (IQR), 61 (28–74)	6 (54.5)	0	0	0	5 (45.4)	-	2 (18.2)	4 *H. influenzae* 1 *Adenovirus* 1 *Influenza A* virus 1 *K. pneumoniae*	5 Third Generation Cephalosporins 2 Beta-Lactam/Beta-Lactamase Inhibitors	RT-PCR via nasopharyngeal and oropharyngeal swabs and sputum specimens	8	Nasopharyngeal and oropharyngeal swabs and sputum specimens were also tested for 33 respiratory pathogens.
Ramadan et al., 2020 [21], Egypt	Prospective cohort, multi- center	260	28 (10.8)	Most common age range was between 51 and 70 years (36.2%)	144 (55.4)	60 (23)	8 (13.3)	24 (40)	28 (10.8)	5 (1.9)	-	5 *S. aureus* 2 *S. pneumoniae* 1 *E. faecalis* 12 *K. pneumoniae* 7 *A. baumannii* 4 *E. coli* 4 *P. aeruginosa* 2 *Enterobacter cloacae* 3 *Candida albicans* 2 *Candida glabrata*	28 Macrolides	Respiratory and blood cultures	7	Eight (28.6%) patients who had co-infections were moderate cases, while 20 (71.4%) were detected in severe COVID-19 patients. Mortality in 25% of SARS-CoV-2 patients was due to co-infections and increased SARS-CoV-2 severity and complications were observed in co-infected patients. Bacterial co-infection and multidrug resistance among patients with COVID-19 in Upper Egypt is common.
Richardson et al., 2020 [38], United States	Retrospective case series, multi-center	1996	42 (2.1)	Not reported	Not reported	Not reported	Not reported	Not reported	Not reported	-	Not reported	22 *Enterovirus*/*Rhinovirus* 7 Coronavirus (non–COVID-19) 4 RSV 3 *Parainfluenza* 3 2 *C. pneumoniae* 2 HMPV 1 *Influenza A* virus 1 *M. pneumoniae*	Not reported	Respiratory viral panel	8	Most patients were obese (60.7% had a BMI≥30) and old (median (IQR): 63 (52–75)).
Rutsaert et al., 2020 [39], Belgium	Retrospective case series, single center	34	6 (17.6)	Median (IQR), 74 (38–86)	6 (100)	6 (100)	6 (100)	4 (66.7)	-	6 (17.6)	-	5 *Aspergillus fumigatus* 1 *Aspergillus flavus*	5 Antifungals	Culture from respiratory specimens and GM detection in the BAL and serum	6	Patients were old and had deteriorating outcomes due to many medical conditions and risk factors.
Schirmer et al., 2021 [13], United States	Retrospective cohort, multi-center	3757	56 (1.5)	Median (IQR), 68 (56–74)	55 (98)	10 (26)	Not reported	10 (18)	1 (0.03)	-	55 (1.5)	2 *Adenovirus* 1 *C. pneumoniae* 13 Coronaviruses (HKU1, NL63, 229E, & OC43) 3 HMPV 2 *Parainfluenza* virus 4 12 *Influenza A* virus 3 *Influenza B* virus 4 RSV 19 *Rhinovirus*/*Enterovirus*	Not reported	Molecular and/or viral culture respiratory assays [multiplex respiratory pathogen panels]	6	Individuals with COVID-19 co-infection had higher odds of being male.
Sepulveda et al., 2020 [61], United States	Retrospective cohort, multi- center	4185	159 (3.8)	Not reported	Not reported	Not reported	Not reported	Not reported	156 (3.7)	3 (0.07)	-	39 *Staphylococcus epidermidis* 28 *Staphylococcus hominis* 8 *E. coli* 8 *Staphylococcus haemolyticus* 8 CoNS 5 *Corynebacterium* 5 *Enterobacter cloacae* complex 5 *Micrococcus luteus* 5 *Staphylococcus warneri* 1 *Actinomyces turicensis* 1 *Aerococcus urinae* 1 *Candida glabrata* 1 *Comamonas estosterone* 1 *Dolosigranulum pigrum* 1 *Eneterobacter* 1 *Enterococcus faecium*, Vancomycin-Resistant 1 *Globicatella sanguinis* 1 *Granulicatella adiacens* 1 *Kocuria marina* 1 *Moraxella osloensis* 1 *Rothia aeria* 1 *S. aureus* 1 *Staphylococcus auricularis* 1 *Staphylococcus lugdunensis* 1 *Streptococcus intermedius* 1 *Streptococcus sanguinis* 2 *Enterococcus faecalis* 2 *E. coli* 2 *Fusobacterium* spp. 2 *Lactobacillus* 2 *Streptococci*, *Viridans* Group 2 *Streptococcus anginosus* 2 *Streptococcus* spp. 6 *K. pneumoniae* 6 MSSA 11 *Staphylococcus capitis* 10 Methicillin Susceptible- CoNS 9 *Bacillus* non-anthracis 7 Methicillin Resistant-CoNS 4 MRSA 3 *Candida albicans*	Not reported	Blood cultures	6	Rate of bacteremia was significantly lower among COVID-19 patients (3.8%) than among COVID-19-negative patients (8.0%) (*p* < 0.001). More than 98% of all positive cultures were detected within 4 days of incubation. The most common causes of true bacteremia among COVID-19 patients were *E. coli* (16.7%), *S. aureus* (13.3%), *K. pneumoniae* (10.0%), and *Enterobacter cloacae* complex (8.3%).
Singh et al., 2021 [62], United States	Retrospective cohort, multi-center	4259	1,558 (36.59)	Mean (SD), 45.21 (20.43)	692 (44.4)	Not reported	Not reported	Not reported	517 (12.1)	-	53 (1.2)	53 *H. influenzae* 75 *S. aureus* 1 *Bordetella pertussis* 1 *C. pneumoniae* 11 *K. pneumoniae* 1 *M. pneumoniae* 49 *S. pneumoniae* 2 *Adenovirus* 1 Coronavirus 1 *Herpes* virus 5 12 EBV 1 RSV 3 *Rhinovirus* 1 HSV 1 HMPV 1 PIV 1 *Influenza* virus	Not reported	RT-PCR for respiratory specimens	6	Co-infections were significantly higher in the older age group (60+ years).
Song et al., 2020 [63], China	Retrospective cohort, single center	89	18 (20.2)	Median (IQR), 35.5 (15–76)	Not reported	2 (11.1)	Not reported	Not reported	18 (20.2)	-	-	6 *K. pneumoniae* 5 *E. coli* 4 *M. catarrhalis* 4 *H. influenzae* 2 *A. baumannii* 2 *S. aureus* 1 *P. aeruginosa* 1 *Streptococcus* Group A	Not reported	RT-PCR for respiratory specimens	6	Authors did not detect co-infection of SARS-CoV-2 with other viruses.
Sun et al., 2020 [40], China	Retrospective case series, single center	36	≈23 (62.86)	Mean (range), 6.43 months (2–12 months)	22 (61.11)	1 (2.78)	1 (2.78)	1 (2.78)	1 (2.8)	-	1 (2.8)	1 *M. pneumonia* 1 *Influenza A* virus	15 Second Generation Cephalosporins15 Macrolides	Unspecified	6	Co-infections were common in infants with COVID-19, which were different from adults with COVID-19; however, authors never provided details of all co-pathogens.
Tagarro et al., 2021 [41], Spain	Retrospective case series, multi-center	41	2 (4.8)	Mean (range), 1 (0–15)	Females were > males	4 (9.7)	1 (2)	0	-	-	2 (4.9)	2 *Influenza B* virus	Not reported	Unspecified	7	Most patients who tested positive for SARS-CoV-2 had no comorbidities (67%).
Tang et al., 2021 [64], China	Retrospective cohort, single center	78	11 (14.1)	Mean (SD), 42.7 (14.9)	41 (52.6)	2 (18.2)	2 (18.2)	0	6 (7.7)	-	6 (7.7)	5 *M. pneumoniae* 4 RSV 2 *C. pneumoniae* 1 *Influenza B* virus 1 *Adenoviruses* 1 *Legionella pneumophila*	48 Fluoroquinolones 5 Beta-Lactam/Beta-Lactamase Inhibitors 3 Linezolid 1 Vancomycin 3 Carbapenems	Serum antibody test (IgM)	6	SARS-CoV-2 patients with co-infections had significantly higher levels of procalcitonin compared to SARS-CoV-2 patients with no co-infections (*p* = 0.002).
Thelen et al., 2021 [65], The Netherlands	Retrospective cohort, multi-center	678	61 (9)	Median (IQR), 70 (58–78)	443 (65.1)	6 (0.9)	Not reported	Not reported	61 (9)	-	-	2 *E. coli* 1 *K. pneumoniae* 1 *P. aeruginosa* 2 *S. pneumoniae* 1 Other *Streptococcus* spp. 1 *S. aureus* 55 CoNS 1 *Corynebacterium* spp.	Not reported	RT-PCR for respiratory specimens AND blood cultures	6	Prevalence of co-infection in SARS-CoV-2 patients was very low compared to *influenza* patient group.
Van Arkel et al., 2020 [42], The Netherlands	Retrospective case series, single center	31	6 (19.3)	Median (IQR), 62.5 (43–83)	6 (100)	6 (100)	6 (100)	4 (66.7)	-		-	5 *Aspergillus fumigatus*	6 Antifungals	Culture from respiratory specimens and GM detection in the BAL, tracheal aspirates, and serum.	6	Pulmonary aspergillosis co-infections occurred after a median of 11.5 days (8–42) after COVID-19 symptom onset and at a median of 5 days (3–28) after ICU admission.
Wang L et al., 2021 [22], United Kingdom	Retrospective cohort, multi- center	1396	37 (2.7)	Median (IQR), 76 (64–82)	28 (75.7)	11 (29.7)	Not reported	10 (27)	37 (2.7)	4 (0.3)	-	12 *E. coli* 2 *K. pneumoniae* 2 *Klebsiella variicola* 4 *Proteus mirabilis* 2 *P. aeruginosa* 1 MRSA 7 MSSA 1 *Staphylococcus epidermidis* 1 *Candida albicans* 2 Group A *Streptococcus* 1 *H. influenzae* 3 *Candida* spp. 2 *Enterococcus faecalis* 3 *S. pneumoniae* 1 *Serratia* spp. 1 *Klebsiella oxytoca* 1 *Streptococcus anginosus* 1 *Bacteroides ovatus* 1 *Granulicatella adiacens* 1 *S. aureus*	Not reported	Unspecified	7	ICU admission and mortality were not different in SARS-CoV-2 patients with co-infections compared to SARS-CoV-2 patients without co-infections [215 (15.8%) vs. 11 (29.7%), *p* = 0.075] and [410 (30.2%) vs. 10 (27.0%), *p* = 0.68], respectively. Bacterial co-infection was infrequent in hospitalized COVID-19 patients within 48 hours of admission.
Wang R et al., 2020 [43], China	Retrospective case series, single center	118	35 (29.7)	Mean (SD), 38.76 (13.79)	(56.8)	19 (16.1)	4 (3.4)	0	35 (29.7)	-	1 (0.8)	40 *M. pneumoniae* 1 *Adenovirus* 1 *Influenza B* virus 1 *Influenza A* virus	Seventy-nine patients received antibiotics; however, agents were not reported.	Serum Antibody test (IgM)	6	Old age, chronic underlying diseases, and smoking history may be risk factors that worsen SARS-CoV-2 disease.
Wang Y et al., 2020 [44], China	Retrospective case series, single center	55	4 (7.3)	Median (IQR), 49 (2–69)	22 (40)	0	0	0	3 (12.7)	-	1 (1.8)	1 EBV 3 *M. pneumoniae*	Not reported	Serologically	7	All patients included in this study had laboratory-confirmed positive results for SARS-CoV-2 and were asymptomatic.
Wang Z et al., 2020 [45], China	Retrospective case series, single center	29 sputum 28 blood	5 (17.2) 4 (14.3)	Majority (51%) were in the range (30–49)	Females were > males	Not reported	Not reported	5 (7.5)	5 (≈17.2)	2 (6.9)	2 (7.1)	2 *Candida albicans* 2 *Enterobacter cloacae* 1 *A. baumannii* 2 *Chlamydia* 1 RSV 1 *Adenovirus*	39 Fluoroquinolones 8 Antifungals	Serum Antibody test (IgM, IgG)	7	Source of patients’ samples tested for co-pathogens were sputum and blood.
Wee et al., 2020 [66], Singapore	Prospective cohort, single center	431	6 (1.4)	Mean (SD), 29.2 (1.7)	6 (100)	0	0	0	0	-	6 (1.4)	3 *Rhinovirus* 2 *Parainfluenza* 1 Other coronavirus (229E/NL63/OC43)	Not reported	RT-PCR for respiratory specimens	6	Co-infections in patients with SARS-CoV-2 shown no increase in morbidity or mortality. All cases of COVID-19 co-infections were young, healthy, and had no medical comorbidities.
Wu C et al., 2020 [67], China	Retrospective cohort, single center	173	1 (0.6)	Majority (80.1%) had a median age <65	Males were > females	53 (26.4)	67 (33.3)	44 (21.9)	-	-	1 (0.6)	1 *Influenza A* virus	Not reported	RT-PCR for respiratory specimens [viruses] AND sputum culture [bacteria and fungi]	8	Most (n = 173 [86.1%]) patients were tested for 9 additional respiratory pathogens. Bacteria and fungi cultures were collected from 148 (73.6%) patients.
Wu Q et al., 2020 [46], China	Retrospective case series, multi-center	34	19 (55.9)	Range (≤3 month to >10 years)	Males were > females	0	1 (2.9)	0	16 (47)	-	10 (29.4)	16 *M. pneumoniae* 2 RSV 2 EBV 3 CMV 1 *Influenza A* virus 1 *Influenza B* virus	15 Macrolides	Unspecified	7	Nearly one-half of the infected children had co-infection with other common respiratory pathogens.
Xia et al., 2020 [47], China	Retrospective case series, single center	20	8 (40)	Range (<1 month to >6 years)	Males were > females	0	0	0	4 (20)	-	5 (25)	1 CMV 2 *Influenza B* virus 1 *Influenza A* virus 4 *Mycoplasma* 1 RSV	Not reported	Unspecified	5	Procalcitonin increased in most of the cases (80%).
Yang et al., 2020 [48], China	Retrospective case series, single center	52	7 (13.5)	Majority (73%) were in the range (50–79)	Males were > females	52 (100)	37 (71)	32 (61.5)	4 (7.7)	3 (5.8)	-	2 *K. pneumoniae* 1 *Aspergillus flavus* 1 *Aspergillus fumigatus* 1 *P. aeruginosa* 1 *Serratia marcescens* 1 *Candida albicans*	Forty-nine patients received antibiotics; however, agents were not reported.	Respiratory and blood cultures	8	Those isolated pathogens caused hospital-acquired infections.
Yue et al., 2020 [68], China	Retrospective cohort, single center	307	176 (57.3)	Mean (SD), 60.3 (16.5)	75 (42.6)	Not reported	Not reported	Not reported	-	-	176 (57.3)	153 *Influenza A* virus 23 *Influenza B* virus	None	Serum antibody test (IgM)	6	Patients co-infected with SARS-CoV-2 and *Influenza B* virus developed poor outcomes (30.4% vs. 5.9%).
Zha et al., 2020 [82], China	Retrospective case series, single center	874	22 (2.5)	Median (IQR), 56.5 (52.5–66.5)	11 (50)	Not reported	Not reported	1 (4.5)	22 (2.5)	-	-	22 *M. pneumoniae*	18 Fluoroquinolones 11 Cephalosporins 3 Beta-Lactam/Beta-Lactamase Inhibitors	RT-PCR for respiratory specimens OR serum antibody test (IgM)	6	Length of cough was longer in the *M. pneumoniae* co-infection group (20 vs. 16.25, *p* = 0.043), while the length of hospital stay was slightly longer (16 vs. 14, *p* = 0.145).
Zhang et al., 2020 [50], China	Retrospective case series, single center	140	7 (5)	Majority (70%) were > 50	1:1 ratio	Not reported	Not reported	Not reported	5 (3.6)	-	2 (1.4)	5 *M. pneumonia* 1 RSV 1 EBV	Not reported	Serum antibody test (IgM, IgG)	5	No clinical and radiological signs of co-infection caused by these pathogens were identified. Increased procalcitonin (*p* = 0.004) was more commonly observed in severe patients.
Zhao et al., 2020 [69], China	Prospective cohort, multi-center	19	2 (10.5)	Median (IQR), 48 (27–56)	Males were > females	0	0	0	1 (5.3)	-	1 (5.3)	1 *Coxsackie* virus 1 *Mycoplasma*	None	RT-PCR for respiratory specimens AND serum antibody test (IgM)	6	Sample size was very small.
Zheng F et al., 2020 [49], China	Retrospective case series, multi-center	25	6 (24)	Range (1 month to ≥6 years)	Males were > females	2 (8)	2 (8)	0	4 (16)	-	2 (8)	2 *Influenza B* virus 3 *M. pneumonia* 1 *Klebsiella aerogenes*	1 Beta-Lactam/Beta-Lactamase Inhibitors 1 Carbapenems 1 Linezolid	Unspecified	5	Highest incidence of infection occurred in children aged <3 years.
Zheng X et al., 2020 [70], China	Retrospective cohort, single center	1001	4 (0.4)	Mean (SD), 35 (19.6)	1:1	0	0	0	-	-	4 (0.4)	3 *Influenza A* virus 3 *Influenza B* virus	Three patients received antibiotics; however, agents were not reported.	RT-PCR for respiratory specimens	7	Patients with both SARS-CoV-2 and *influenza* virus infection showed similar clinical characteristics to those patients with SARS-CoV-2 infection only. Co-infection of SARS-CoV-2 and *influenza* viruses was low.
Zhu et al., 2020 [4], China	Retrospective cohort, single center	257	243 (94.5)	Median (IQR), 51 (2−99)	138 (53.7)	3 (1.2)	0	0	236 (91.8)	60 (23.3)	81 (31.5)	153 *S. pneumoniae* 143 *K. pneumoniae* 103 *H. influenza* 60 *Aspergillus* 52 EBV 24 *E. coli* 21 *S. aureus* 12 *Rhinovirus* 12 *P. aeruginosa* 11 *M. catarrhalis* 10 *Adenovirus* 8 HSV 7 *A. baumannii* 6 *C. pneumoniae* 6 *Mucor* 5 *Influenza B* 4 *M. pneumonia* 3 *Bordetella pertussis* 2 *Candida* 3 CMV 2 *Influenza A* virus 1 *Bocavirus* 1 HMPV 1 *Cryptococcus*	Not reported	RT-PCR for respiratory specimens	7	Highest and lowest rates of co-infections were found in patients aged 15–44 and below 15, respectively. Most co-infections occurred within 1–4 days of onset of COVID-19 disease. Proportion of viral, fungal and bacterial co-infections were the highest in severe COVID-19 cases.

Abbreviations: BA, bronchoaspirate; BAL, bronchoalveolar lavage; GM, galactomannan; IgG, immunoglobulin G; IgM, immunoglobulin M; RT-PCR, reverse transcription polymerase chain reaction; COVID-19, coronavirus disease 2019; ICU, intensive care unit; MERS-CoV, Middle East respiratory syndrome coronavirus; SARS-CoV-2, severe acute respiratory syndrome coronavirus 2; NOS, Newcastle–Ottawa scale; *C. pneumoniae*, *Chlamydia pneumoniae*; *M. pneumoniae, Mycoplasma pneumoniae*; RSV, Respiratory syncytial virus; *H. influenzae*, *Haemophilus influenzae*; *K. pneumoniae*, *Klebsiella pneumoniae*; EBV, Epstein–Barr virus; *P. aeruginosa*, *Pseudomonas aeruginosa*; HcoV-HKU1, human coronavirus HKU1; *S. pneumoniae*, *Streptococcus pneumoniae*; *M. catarrhalis*, *Moraxella catarrhalis*; ARDS, acute respiratory distress syndrome; MRSA, methicillin-resistant *Staphylococcus aureus*; CMV, *cytomegalovirus*; *S. aureus*, *Staphylococcus aureus*; HSV, herpes simplex virus; *A. baumannii*, *Acinetobacter baumannii*; MSSA, methicillin-susceptible *Staphylococcus aureus*; CoNS, coagulase-negative *staphylococci*; AOR, adjusted odds ratio; CI, confidence interval; HHV6, human herpes virus 6; *E. coli*, *Escherichia coli*; spp., species; HMPV, human metapneumovirus.

**Table 2:** Proportion of all identified SARS-CoV-2 bacterial co-infections (N = 3468).

Bacterial Pathogen Type	Identified Number (%)	Bacterial Pathogen Type	Identified Number (%)
*S. aureus*	1,095 (31.6)	*Corynebacterium* spp.	6 (0.2)
*M. catarrhalis*	352 (10.1)	*Bordetella pertussis*	5 (0.1)
*M. pneumoniae*	338 (9.7)	*Micrococcus luteus*	5 (0.1)
*S. pneumoniae*	316 (9.1)	*Citrobacter koseri*	4 (0.1)
*C. pneumoniae*	261 (7.5)	*Hafnia alvei*	3 (0.1)
*K. pneumoniae*	259 (7.5)	*S. maltophilia*	3 (0.1)
*H. influenzae*	197 (5.7)	*Streptococcus anginosus*	3 (0.1)
CoNS	115 (3.3)	*Streptococcus* Group A	3 (0.1)
*E. coli*	65 (1.9)	*Burkholderia cepacia*	3 (0.1)
*P. aeruginosa*	48 (1.4)	*Bacteroides* spp.	3 (0.1)
*Staphylococcus epidermidis*	42 (1.2)	*Stephanoascus ciferrii*	3 (0.1)
MSSA	31 (0.9)	*Elizabethkingia meningosepticum*	2 (0.1)
Other *Enterococcus* spp.	31 (0.9)	*Granulicatella adiacens*	2 (0.1)
*Staphylococcus hominis*	28 (0.8)	*Lactobacillus*	2 (0.1)
*A. baumannii*	24 (0.7)	*Streptococci agalactiae*	2 (0.1)
*Enterococcus faecium*	23 (0.7)	*Fusobacterium* spp.	2 (0.1)
MRSA	18 (0.5)	*Aerococcus urinae*	1 (0.03)
*Enterococcus faecalis*	17 (0.5)	*Streptococcus intermedius*	1 (0.03)
Other *Klebsiella* spp.	15 (0.4)	*Streptococcus sanguinis*	1 (0.03)
*Enterobacter cloacae*	15 (0.4)	*Actinomyces turicensis*	1 (0.03)
*Pseudomonas* spp.	13 (0.4)	*Providencia* spp.	1 (0.03)
*Streptococcus pneumoniae*	12 (0.3)	*Ralstonia mannitolilytica*	1 (0.03)
*Staphylococcus capitis*	11 (0.3)	*Rothia aeria*	1 (0.03)
Methicillin Susceptible- CoNS	10 (0.3)	*Legionella pneumophila*	1 (0.03)
Other *Streptococcus* spp.	9 (0.3)	*Clostridium perfringens*	1 (0.03)
*Proteus mirabilis*	9 (0.3)	*Comamonas testosteroni*	1 (0.03)
*Bacillus* non-anthracis	9 (0.3)	*Dolosigranulum pigrum*	1 (0.03)
Other *Staphylococcus* spp.	8 (0.2)	*Globicatella sanguinis*	1 (0.03)
*Serratia marcescens*	8 (0.2)	*Kocuria marina*	1 (0.03)
*Staphylococcus haemolyticus*	8 (0.2)	*Morganella morganii*	1 (0.03)
*Stenotrophomonas maltophilia*	8 (0.2)	*Moraxella osloensis*	1 (0.03)
Methicillin Resistant- CoNS	7 (0.2)		

Abbreviations: SARS-CoV-2, severe acute respiratory syndrome coronavirus 2; *C. pneumoniae*, *Chlamydia pneumoniae*; *M. pneumoniae*, *Mycoplasma pneumoniae*; *H. influenzae*, *Haemophilus influenzae*; *K. pneumoniae*, *Klebsiella pneumoniae*; *P. aeruginosa*, *Pseudomonas aeruginosa*; *S. pneumoniae*, *Streptococcus pneumoniae*; *M. catarrhalis*, *Moraxella catarrhalis*; MRSA, methicillin-resistant *Staphylococcus aureus*; *S. aureus*, *Staphylococcus aureus*; *A. baumannii*, *Acinetobacter baumannii*; MSSA, methicillin-susceptible *Staphylococcus aureus*; CoNS, coagulase-negative *staphylococci*; *E. coli*, *Escherichia coli*; spp., species.

**Table 3:** Proportion of all identified SARS-CoV-2 fungal co-infections (N = 192).

Fungal Pathogen Type	Identified Number (%)
*Aspergillus* spp.	68 (35.4)
*Aspergillus fumigatus*	43 (22.4)
Other *Candida* spp.	29 (15.1)
*Candida albicans*	25 (13)
*Aspergillus flavus*	10 (5.2)
*Mucor*	6 (3.1)
*Candida glabrata*	5 (2.6)
*Aspergillus niger*	4 (2.1)
*Aspergillus terreus*	1 (0.5)
*Cryptococcus*	1 (0.5)

Abbreviations: SARS-CoV-2, severe acute respiratory syndrome coronavirus 2; spp., species.

**Table 4:** Proportion of all identified SARS-CoV-2 respiratory viral co-infections (N = 2392).

Respiratory Viral Pathogen Type	Identified Number (%)
EBV	644 (26.9)
HHV6	574 (24)
*Influenza A* virus	355 (14.8)
HMPV	328 (13.7)
*Adenovirus*	144 (6)
*Influenza B* virus	68 (2.8)
*Rhinovirus/Enterovirus*	68 (2.8)
RSV	52 (2.2)
*Parainfluenza* [1, 2, 3 and 4] virus	29 (1.2)
HcoV-OC43	11 (0.5)
*Rhinovirus*	22 (0.9)
*Influenza* virus (H_1_N_1_)	18 (0.8)
HcoV-HKU1	16 (0.7)
HcoV-NL63	13 (0.5)
*Bocavirus*	10 (0.4)
HSV	10 (0.4)
HcoV-229E	9 (0.4)
CMV	8 (0.3)
MERS-CoV	8 (0.3)
*Enterovirus*	1 (0.04)
*Rotavirus*	1 (0.04)
*Coxsackie* virus	1 (0.04)
Human Coronavirus 229E	1 (0.04)
*Herpes* virus 5	1 (0.04)

Abbreviations: SARS-CoV-2, severe acute respiratory syndrome coronavirus 2; RSV, respiratory syncytial virus; EBV, Epstein–Barr virus; HcoV-HKU1, human coronavirus HKU1; CMV, cytomegalovirus; HSV, herpes simplex virus; HHV6, human herpes virus 6; HMPV, human metapneumovirus.

## Data Availability

Data are available upon request. Please contact author for data requests.
